# Large-scale expansion and characterization of CD3^+^ T-cells in the Quantum^®^ Cell Expansion System

**DOI:** 10.1186/s12967-019-2001-5

**Published:** 2019-08-07

**Authors:** Claire Coeshott, Boah Vang, Mark Jones, Brian Nankervis

**Affiliations:** Terumo BCT, Inc., 10810 West Collins Avenue, Lakewood, CO 80215 USA

**Keywords:** Cell expansion, Cell proliferation, Hollow-fiber, Immunotherapy, Quantum, T-cell

## Abstract

**Background:**

The rapid evolution of cell-based immunotherapies such as chimeric antigen receptor T-cells for treatment of hematological cancers has precipitated the need for a platform to expand these cells ex vivo in a safe, efficient, and reproducible manner. In the Quantum^®^ Cell Expansion System (Quantum system) we evaluated the expansion of T-cells from healthy donors in a functionally-closed environment that reduces time and resources needed to produce a therapeutic dose.

**Methods:**

Mononuclear cells from leukapheresis products from 5 healthy donors were activated with anti-CD3/CD28 Dynabeads^®^ and expanded in the Quantum system for 8–9 days using xeno-free, serum-free medium and IL-2. Harvested cells were phenotyped by flow cytometry and evaluated for cytokine secretion by multiplex assays.

**Results:**

From starting products of 30 or 85 × 10^6^ mononuclear cells, CD3^+^ T-cell populations expanded over 500-fold following stimulation to provide yields up to 25 × 10^9^ cells within 8 days. T-cell yields from all donors were similar in terms of harvest numbers, viability and doubling times. Functionality (secretion of IFN-γ, IL-2 and TNF-α) was retained in harvested T-cells upon restimulation in vitro and T-cells displayed therapeutically-relevant less-differentiated phenotypes of naïve and central memory T-cells, with low expression of exhaustion markers LAG-3 and PD-1.

**Conclusions:**

The Quantum system has been successfully used to produce large quantities of functional T-cells at clinical dosing scale and within a short timeframe. This platform could have wide applicability for autologous and allogeneic cellular immunotherapies for the treatment of cancer.

**Electronic supplementary material:**

The online version of this article (10.1186/s12967-019-2001-5) contains supplementary material, which is available to authorized users.

## Introduction

There have been remarkable recent advances in cellular immunotherapies to treat cancer, in particular in the expansion and use of gene-modified T-cells, expressing chimeric antigen receptors (CARs), for the treatment of advanced hematologic cancers. Recent U.S. Food and Drug Administration (FDA) approvals of tisagenlecleucel [1, 2], which targets B-cell acute lymphoblastic leukemia (B-ALL) and relapsed or refractory (r/r) diffuse large B-cell lymphoma (DLBCL), and of axicabtagene ciloleucel (axi-cel) [3], which targets r/r DLBCL, are evidence of the promise of these approaches for the future treatment of cancer. These cellular therapies require the manufacture of up to billions of autologous T-cells for re-infusion to the patient and thus technologies that can address the labor intensity, costs and requirements for Good Manufacturing Practice (GMP) to achieve these cell numbers are being sought.

The Quantum^®^ Cell Expansion System (Quantum system) is a functionally-closed, automated, hollow-fiber bioreactor system that can offer versatility, efficiency and scalability for expansion of gene-modified T cells. Although the Quantum system was designed to grow both adherent and suspension cells, to date it has mainly been used for growth of adherent cells such as mesenchymal stromal cells [also known as mesenchymal stem cells (MSCs)] [4–6] and adipose-derived stem cells [7], and its advantages over manual processes for scale-up of MSCs in compliance with GMP have been highlighted [8]. More recently, the Quantum system has been used to grow T-cells derived from peripheral blood of healthy donors and process improvements were shown to support low seeding densities, more sustained log phase growth and high yields [9].

The operation of the Quantum system has been described in several previous publications [4–6, 9]. In brief, the system consists of a synthetic hollow-fiber bioreactor that is part of a sterile closed-loop circuit for media and gas exchange. The bioreactor and the fluid circuit comprise a single-use disposable set that is mounted onto the Quantum system. The bioreactor itself is composed of 11,520 hollow fibers with a total intracapillary (IC) surface area of 2.1 m^2^ and 124 mL of fluid volume within the fibers of the bioreactor. Typical culture manipulations (such as cell seeding, media exchanges, cell feeding and cell harvest) are managed by the computer-controlled system, which uses pumps and automated valves to direct fluid through the disposable set and to exchange gas with the medium. The functionally-closed nature of the disposable set is maintained through the sterile connection/disconnection of bags that are used for all fluids. Gas control in the system is managed using hollow-fiber technology in a gas transfer module, with gas supplied from a user-provided premixed gas tank.

**Fig. 1:**
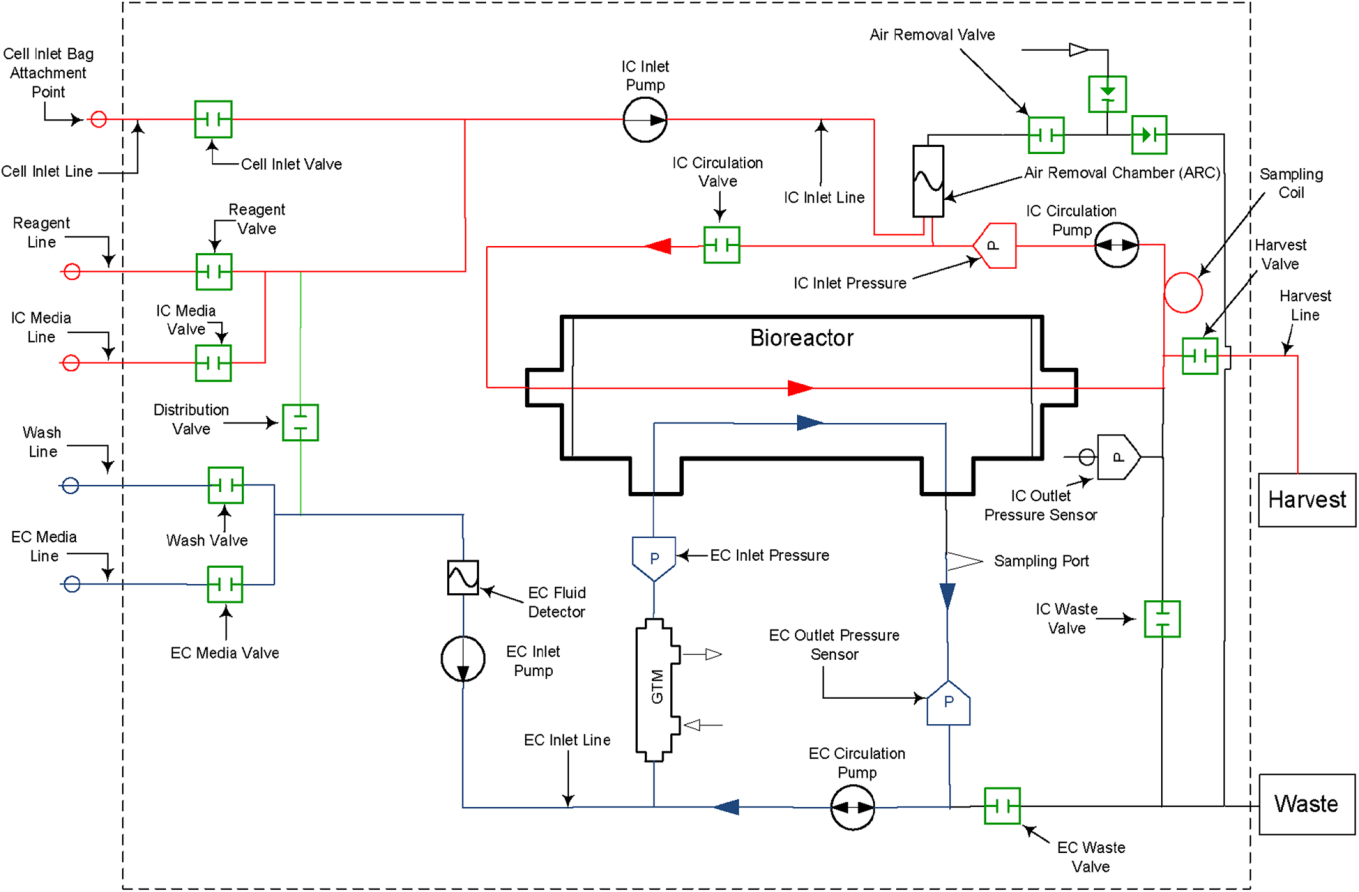
Quantum system hydraulic layout with intracapillary (IC) loop (red) and extracapillary (EC) loop (blue). Copyright Terumo BCT, Inc., 2019. Used with permission. A version of this figure has been previously published [9]

The Quantum system fluid circuit is designed around two fluid loops for the intracapillary (IC) and extracapillary (EC) sides of the bioreactor (Fig. 1). The total volume of the IC fluid circuit is 175 mL (of which 124 mL is the volume within the fibers of the bioreactor) and that of the EC fluid circuit is 305 mL. The bioreactor membrane allows free gas diffusion between the IC and EC sides of the bioreactor, as well as small molecule diffusion, which allows glucose and lactate to freely pass from one side of the membrane to the other. Larger macromolecules are sequestered on the side of the membrane where they are added; for this reason, it is important to ensure that medium with larger molecules critical for cell culture (such as cytokines and growth factors) is loaded on the IC side when cells are expanded on this side of the bioreactor.

In this report, we demonstrate that the Quantum system is a versatile platform for the manufacture of T-cells that retain functionality and that display phenotypes of relevance for cancer immunotherapy. Moreover, we show that this can be done at a large scale, consistently generating billions of CD3^+^ T-cells in a short timeframe, while providing the potential for significant reduction of labor and expense through automated programming and unique dual-loop circuits.

## Materials and methods

### Preparation of peripheral blood mononuclear cells (PBMCs)

Peripheral blood leukapheresis products were obtained from individual healthy donors (AllCells, LLC, Alameda, CA, HemaCare Corp, Van Nuys, CA or Key Biologics LLC, Memphis, TN) using either COBE^®^ Spectra, Spectra Optia^®^ Apheresis System or Trima^®^ Accel Automated Blood Collection System (Terumo BCT, Lakewood, CO). PBMCs were further purified to remove red blood cells and platelets by Ficoll^®^ density gradient separation. PBMCs were subsequently cryopreserved with Cryostor^®^ CS10 (BioLife Solutions Inc., Bothell, WA) in liquid nitrogen vapor phase until used in expansion studies.

### Expansion of T-cells in the Quantum system

PRIME-XV T-cell Expansion XSFM medium, a complete xeno-free, serum-free medium, was used for all T-cell expansions (FUJIFLM Irvine Scientific, Irvine, CA). This medium was additionally supplemented where indicated with recombinant human interleukin 2 (IL-2) improved sequence (Miltenyi Biotec GmbH, Germany) at a final concentration of 100 IU/mL. PBMCs were combined with anti-CD3/anti-CD28 DynaBeads^®^ (Gibco, Waltham, MA) in bead: PBMC ratios of 3:1 (low cell seeding) or 2:1 (high cell seeding) and then mixed at room temperature (RT) for 10 minutes (min) using a rotating cell mixer at a concentration of 4 × 10^6^ to 6 × 10^6^ cells/mL. Cells were seeded at either 30 × 10^6^ PBMCs per Quantum system (low seeding) or 85 × 10^6^ PBMCs per Quantum system (high seeding). Cell-bead mixtures were diluted to 50 mL medium without IL-2 and added to a cell inlet bag (Terumo BCT), then loaded into the IC loop of the Quantum system. The day of seeding is referred to as Day 0 of the expansion. Cell expansion methods in the Quantum system were as described previously [9] with the following modifications.

Medium supplemented with IL-2 was initially added to the cultures via continuous perfusion at 0.1 mL/min to the IC loop. Perfusion rate to the IC loop increased incrementally up to 0.4 mL/min to maintain lactate concentration levels below 15 mmol/L. To retain the cells in the fibers of the bioreactor, IC medium was directed into both the inlet and the outlet of the bioreactor. This was accomplished by directing the IC circulation pump in the opposite (or negative) direction of the IC inlet flow, at half the flow rate. Further details of the custom tasks and settings for the Quantum system are shown in Additional file 1. Additional feed rate increases required to supply gas and remove lactate from the culture were accomplished by EC perfusion with medium without IL-2, reaching a maximum flow rate of 2.8 mL/min. Cell mixing was initiated on Day 2 of culture by circulating the IC contents at a rate of 300 mL/min for 4 min. Cells were then moved from the IC loop and positioned back in the bioreactor using an IC inlet rate of 80 mL/min, with an IC circulation rate of (−) 40 mL/min to create a bidirectional reseeding into the bioreactor. Cell mixing occurred twice per day once EC perfusion was required.

Cell samples were obtained in process from the IC loop beginning on Day 6 of expansion by pausing the cell mixing step 30 s before completion and removing a 6-in. length of tubing from the IC sample coil using a TSCD^®^-Q Sterile Tubing Welder (Terumo BCT). Lactate and glucose measurements obtained from the EC sample port were recorded by i-STAT handheld blood analyzer (Abbott Point of Care, Princeton, NJ) starting on Day 3.

### Quantum system T-cell harvest

Cells were harvested from the Quantum system using an automated custom task. To facilitate this method of harvest, the cell suspension was circulated on the IC side of the bioreactor for 4 min at a rate of 300 mL/min with the bioreactor in motion. Further details of the harvest tasks are shown in Additional file 1. After harvest, cells were assessed for viability and cell yield, and then cryopreserved in liquid nitrogen vapor phase until assayed for phenotype and cytokine secretion.

Cell harvest numbers and viability were measured by Vi-CELL™ XR automated cell counter (Vi-CELL, Beckman Coulter^®^, Indianapolis, IN), using trypan blue dye exclusion. The device was used according to manufacturer’s instructions. Cells were cryopreserved by resuspension in Cryostor CS10 at a final concentration of 20 × 10^6^ cells/mL until assayed.

CD3^+^ T-cell counts were obtained from flow cytometry (see below) and from Vi-CELL (total cells × % CD3^+^ T-cells) and were used to calculate population doublings (PD), doubling times (dT), and fold-expansion (FE) during culture according to the following formulae:

$${\text{PD }} = { \ln }{{\left( {{{{\text{CD3}}^{+} \;\;{\text{T{-}cells}}\;\;{\text{harvested}}} / {{\text{CD3}}^{ + } \;\;{\text{T{-}cells}}\;\;{\text{seeded}}}}} \right)} / {{ \ln }\left( 2\right)}},$$

$${\text{dT}} = ({ \ln }\left( 2\right) \times t \times { 24})/{ \ln }\left( {{\text{CD3}}^{ + } \;\;{\text{T{-}cells}}\;\;{\text{harvested}}/{\text{CD3}}^{ + } \;\;{\text{T{-}cells}}\;\;{\text{seeded}}} \right),$$

$${\text{FE }} = {\text{ CD3}}^{ + } \;\;{\text{T{-}cells}}\;\;{\text{harvested}}/{\text{CD3}}^{ + } \;\;{\text{T{-}cells}}\;\;{\text{seeded}},$$

where *t* is the number of days in culture.

### Flow cytometry

Flow cytometry was performed at the Human Immune Monitoring Shared Resource, University of Colorado School of Medicine, Aurora, CO. Thawed T-cells were counted using trypan blue and 1 × 10^6^ cells were incubated with blocking buffer (PBS/10% human serum/10% mouse serum) in a 96-well plate for 10 min at RT. Cells were washed (PBS/2% fetal bovine serum) and incubated with directly-conjugated antibodies with the following specificities and fluorescent labels: CD3 BV786 (UCHT1), CD4 BUV395 (SK3), CD8 BUV737 (SK1), CD45RO APC-eFluor780 (UCHL1), CCR7 BV421 (150503), (all from BD Biosciences, San Jose, CA); CD45RA PE-Cy5 (HI100), Tim-3 BV510 (F382E2), LAG-3 BV650 (11C3C65) and PD-1 BV711 (EH122H7) (all from BioLegend, San Diego, CA). Following staining and washing, samples were fixed in 1% paraformaldehyde. Fixable Red Dead Cell staining kit (Thermo Fisher Scientific, Waltham, MA) was used to evaluate viability. Data were collected on a BD LSRFortessa X-20 and analyzed using FlowJo^®^ V10 software. A control cell sample consisting of T-cells separated from healthy donor PBMCs using a T-cell negative selection kit (Miltenyi Biotec) was used as a comparator for both flow and cytokine secretion assays.

### Cytokine secretion assay

Interferon-γ (IFN-γ), IL-2 and tumor necrosis factor-α (TNF-α) concentrations were measured using multiplex cytokine arrays [V-PLEX, Meso Scale Discovery (MSD), Meso Scale Diagnostics, Rockville, MD] according to the manufacturer’s instructions. Assays were performed by the Human Immune Monitoring Shared Resource, University of Colorado School of Medicine, Aurora, CO. Briefly, thawed and washed T-cells were plated at 2 × 10^5^ viable cells/well in 96-U well plates and incubated for 6 h in a 37 °C incubator with 5% CO_2_ prior to addition of Dynabeads (anti-CD3/anti-CD28; 2 µL/well). Cells were stimulated with beads for 18 h, then supernatants were harvested and stored at − 80 °C until assay. All assays included the T-cell comparator control described above for flow cytometry assays.

Pre-coated V-PLEX plates (MSD) were washed using an automated plate washer (BioTek ELX5012), and calibrators or thawed, diluted supernatants were added, followed by further incubation for 2 h at RT on a Compact Digital Microplate shaker (Thermo Fisher Scientific) at 600 rpm. Plates were again washed, and detection antibodies were added and incubated for 2 h at RT. After washing, 2× Read Buffer (MSD) was added and plates were immediately read on a MesoQuickPlex SQ120 electrochemiluminescent plate reader. Data were analyzed using Workbench software (MSD).

### Statistical analysis

Descriptive statistics showing means, standard deviations and coefficients of variation (CV) for cell expansion were calculated using Microsoft^®^ Excel^®^ data analysis.

## Results

### T-cell expansion characteristics in the Quantum system

**Fig. 2:**
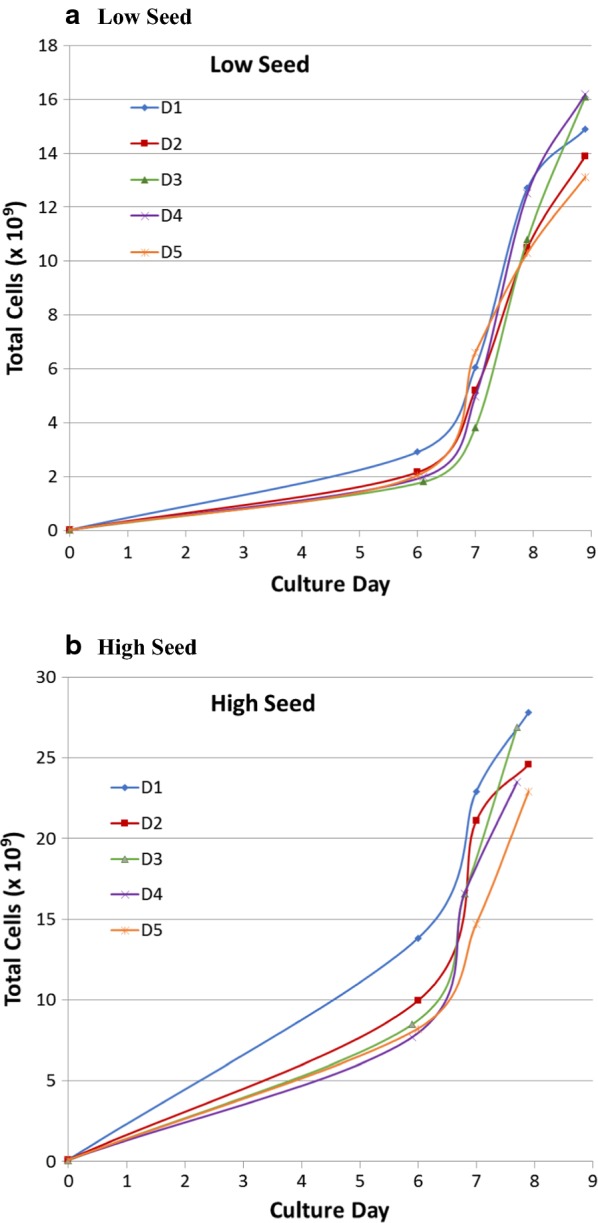
Growth curves for T-cells expanded in the Quantum system. PBMCs were incubated with anti-CD3/CD28 DynaBeads then seeded in the Quantum system bioreactor at either 85 × 10^6^ (**a**) or 30 × 10^6^ (**b**) PBMCs. Five individual donors are shown. Numbers of cells indicates total mononuclear cells at various timepoints enumerated from samples taken from the IC loop following mixing of the entire IC compartment. Final cell numbers are for products enumerated following automated harvest from the Quantum system. *D* donor

PBMCs from 5 healthy donors were seeded in the Quantum system at either 30 × 10^6^ or 85 × 10^6^ viable cells (low and high seeding respectively) and harvested 8 days (high seed) or 9 days (low seed) later. Low and high seeding numbers of PBMCs were used in order to investigate the versatility of the Quantum system to expand T-cell products from a range of starting CD3^+^ T-cell numbers. The expansion kinetics for cells from individual donors are shown in Fig. 2 and the growth characteristics are summarized in Table 1.

**Table 1:** Expansion of T-cells in the Quantum system

Donor	PBMCs seeded (×10^6^)	CD3^+^ T-cells seeded (%)	Total cells harvested (×10^9^)	Viability (%)	CD3^+^ T-cells harvested (%)	CD3^+^ T- cell fold-expansion	CD3^+^ T-cell doubling time (h)	CD3^+^ T-cells population doublings	Cell culture concentration at harvest (×10^6^ cells/mL)^a^
1	30	36.9	14.9	91.9	94.2	1268	20.7	10.3	120
2		46.6	13.9	92.5	96.1	956	21.6	9.9	112
3		55.0	16.1	93.8	97.5	951	21.6	9.9	130
4		32.5	16.2	94.0	96.0	1595	20.1	10.6	131
5		23.6	13.1	92.5	96.6	1787	19.8	10.8	106
Mean		38.9	14.8	93.7	96.1	1311	20.7	10.3	120
SD		12.2	1.3	2.0	1.2	376	0.8	0.4	11
% CV		31.4	9.1	2.1	1.3	29	4.0	4.1	9.1
1	85	36.9	27.8	91.0	91.9	815	19.6	9.7	224
2		46.6	24.6	92.0	94.3	586	20.6	9.2	198
3		55.0	26.9	94.0	94.3	543	20.3	9.1	217
4		32.5	23.5	93.0	92.7	789	19.2	9.6	190
5		23.6	22.9	90.3	94.5	1079	18.8	10.1	185
Mean		38.9	25.1	92.1	93.5	762	19.9	9.5	203
SD		12.2	2.1	1.5	1.2	214	0.8	0.4	17
% CV		31.4	8.5	1.6	1.3	28	4.2	4.1	8.5

Fold expansion, doubling times and population doublings were calculated as described in “Materials and methods”

^a^Total cell concentration at harvest was calculated from the total cells harvested and the volume within the fibers of the bioreactor (124 mL)

With low seeding, the mean cell yield was 14.8 × 10^9^ total cells (range 13.1 × 10^9^ to 16.2 × 10^9^ cells), with a mean of 96.1% (range 94.2% to 97.5%) CD3^+^ T-cells in the harvested products. For high cell seeding, mean yield was 25.1 × 10^9^ total cells (range 22.9 × 10^9^ to 27.8 × 10^9^ cells) with a mean of 93.5% (range 91.9% to 94.5%) CD3^+^ T-cells. All cell products had high viability, with a mean of 92.5% (range 90.3% to 94.0%). The mean fold CD3^+^ T-cell expansion was 1311 (range 951 to 1787) and 762 (range 543 to 1079) for low and high seed cultures respectively. Doubling times for CD3^+^ T-cells in low seed and high seed cultures were very similar, with a mean of 20.7 h (range 19.8 h to 21.6 h) for low seed and a mean of 19.9 h (range 18.8 h to 20.6 h) for high seed. CD3^+^ T-cell total population doublings were also very similar among the cell products ranging from 9.9 to 10.8 (mean 10.3) for low seed and 9.1 to 10.1 (mean 9.5) for high seed.

**Fig. 3:**
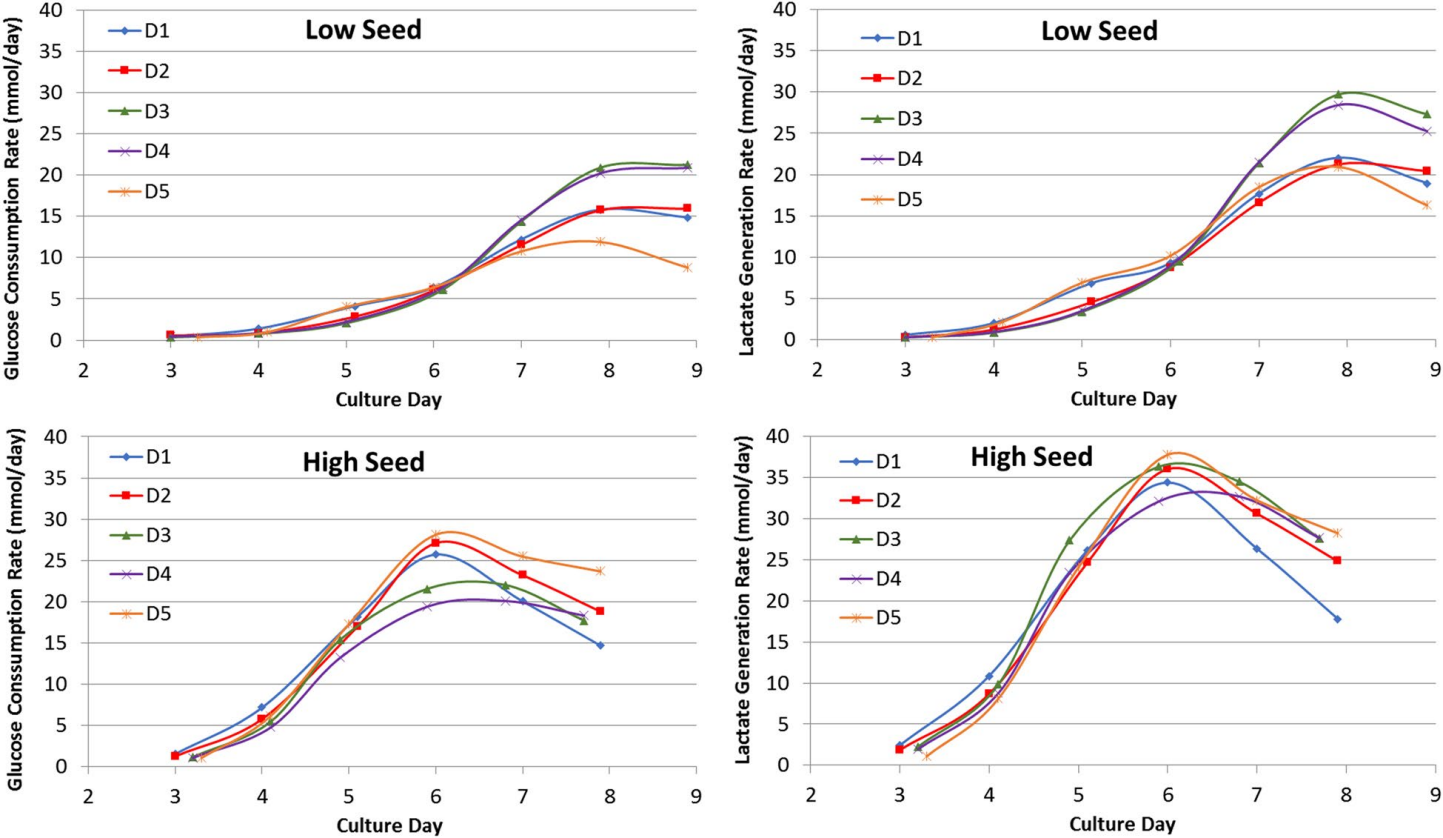
Lactate generation rates and glucose consumption rates for T-cells expanded in the Quantum system

Glucose consumption and lactate production rates were monitored daily starting at Day 3 and are shown in Fig. 3. More exaggerated peaks for lactate generation rates were seen for high seed cultures at Day 6 followed by a more rapid decline than that seen for low seed cultures. Cells from donors 3 and 4 had higher glucose consumption and lactate production than cells from other donors in low seed cultures.

### Phenotypes of T-cell products

**Fig. 4:**
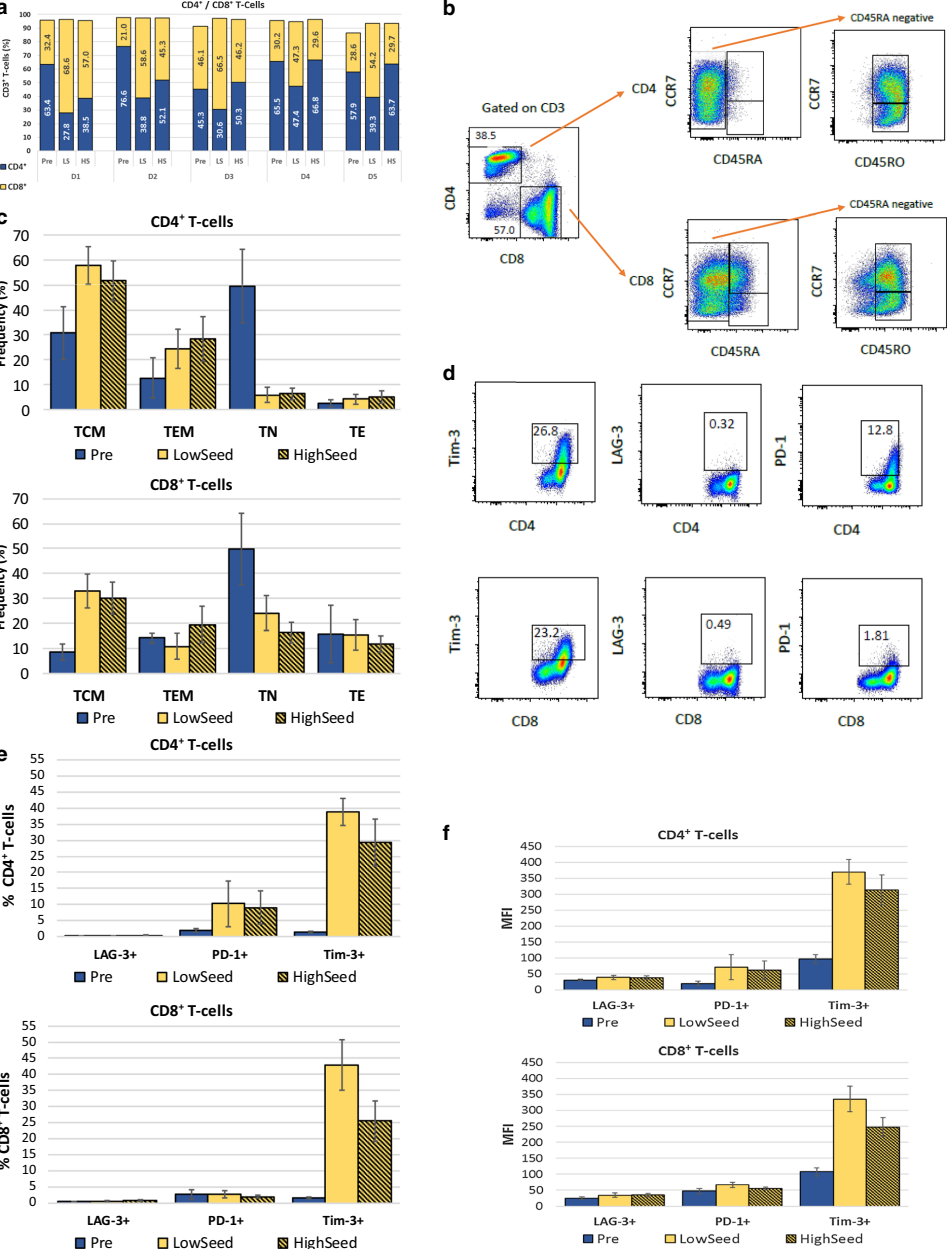
Phenotypes of T-cells expanded in the Quantum system. **a** CD4^+^/CD8^+^ T-cell ratios. Percentages of the CD3^+^ T-cell parent population are shown. Cell populations are shown for pre-seeding (pre) and after harvest (post) for T-cells expanded from PBMCs of 5 healthy donors. **b** Gating strategy used to identify naive and memory T-cell phenotypes. One representative example is shown (donor 1, high seed). **c** Frequencies within the CD4^+^ and CD8^+^ T-cell populations of TN (CD45RA^+^/CCR7^+^), TCM (CD45RO^+^/CCR7^+^), TEM (CD45RO^+^/CCR7^−^) and TE (CD45RA^+^/CCR7^−^) subsets. Data are expressed as % of CD4^+^ or CD8^+^ T-cell populations. **d** Gating strategy used to analyze the expression of exhaustion markers LAG-3, PD-1 and Tim-3 on CD4^+^ and CD8^+^ T-cells. One representative example is shown (donor 1). **e** Frequencies of CD4^+^ and CD8^+^ T-cells expressing exhaustion markers. **f** MFI for expression of exhaustion markers on positively-gated events for CD4^+^ and CD8^+^ T-cells. Error  bars represent one standard deviation from the mean. *Pre* pre-expansion PBMCS, *LS* low seed, *HS* high seed

Harvested products and PBMCs pre-expansion from all donors were evaluated by flow cytometry for the following T-cell surface markers: CD3, CD4, CD8, CD45RA, CD45RO, CCR7, LAG-3, PD-1 and Tim-3. As shown in Fig. 4a, for cells expanded from low seeding numbers, cells from 4 of 5 donors exhibited preferential expansion of CD8^+^ T-cell populations in the Quantum system with 54.2% to 68.6% of the harvested CD3^+^ T-cell population, whereas T cells from one donor had a more equal balance of CD4^+^ and CD8^+^ T-cells (47% for each sub-population). In contrast to the low seed cell products, only T-cells from one donor (donor 1) maintained preferential expansion of CD8^+^ T-cells (57.0%) in a high seed culture. T-cells from two donors (donors 2 and 3) displayed balanced CD4^+^:CD8^+^ T-cell ratios, in contrast to their low seed expansions, and T-cells from 2 other donors (donors 4 and 5) had more exaggerated skewing to CD4^+^ T-cells from high seeding numbers, with 68.5% and 63.7% CD4^+^ T-cells, respectively. The difference between low and high seeding outcomes was not a facet of the starting cell population, since both expansions were initiated from the same starting populations as shown in Table 1.

The distribution of CD4^+^ and CD8^+^ naïve, memory and effector T-cell subsets was assessed by flow cytometry using a gating strategy illustrated in Fig. 4b. Using this approach, CD4^+^ and CD8^+^ T-cells were found to have different distributions of naïve, memory and effector phenotypes (Fig. 4c) but with only small or negligible differences between low and high seeding expansions. For CD4^+^ T-cells, the dominant fraction was of the central memory phenotype (TCM; CD45RO^+^/CCR7^+^), ranging from 43.5 to 63.7% of the harvested CD4^+^ T-cell populations, whereas effector memory T-cells (TEM; CD45RO^+^/CCR7^−^) were present at lower frequencies (14.4% to 39.5%). Naïve T-cells (TN; CD45RA^+^/CCR7^+^) and effector T-cells (TE; CD45RA^+^/CCR7^−^) were represented at less than 10% of CD4^+^ T-cells for all harvested cell products and TN were greatly reduced compared to the starting cell populations. For CD8^+^ T-cells, there was more heterogeneity in naïve/memory phenotypes among the harvested products with some differences between the low and high seeding expansions. TCM frequencies ranged from 25.4 to 40.7% for low seed and 22.7 to 38% for high seed and only two donors had a predominant population of TCM (donors 1 and 3). TN were also well-represented in the harvested CD8^+^ T-cells of 3 donors (at 25.7%, 29.7%, 31.4% in products from low seed) with a range of 16.4% to 31.4% TN for low seed and 11.0% to 20.5% TN for high seed. CD8^+^ TEM and TE were generally present at lower frequencies with all TEM frequencies at less than 20% for low seed and from 7.6 to 26.8% for high seed products. TE frequencies ranged from 8.1 to 24.5% for low seed and from 6.0 to 14.2% for high seed.

Potential T-cell exhaustion was assessed in the harvested cell products by the expression of PD-1, LAG-3 and Tim-3 (Fig. 4d, e). The proportions of CD4^+^ and CD8^+^ T-cells expressing LAG-3 were very low in all harvested T-cell products (< 1%) and showed negligible increases following expansion. The proportion of CD4^+^ PD-1^+^ T-cells increased moderately during expansion from an average of 2% pre-expansion to an average at harvest of 9.1% (range 2.0% to 13.4%) for high seed and of 10.3% (range 2.5% to 21.1%) for low seed. For CD8^+^ T-cells, there was no appreciable increase in frequency of PD-1 expression on harvested cell products with all products having less than 5% PD-1^+^ CD8^+^ T-cells. The frequency of Tim-3-expressing T-cells increased for all donors compared to pre-expansion PBMC populations for both CD4^+^ and CD8^+^ T-cell products although the frequency was lower from harvests of high seed cultures. For CD4^+^ T-cells, Tim-3^+^ cells increased from an average of 1.4% (range 1.1% to 1.7%) pre-expansion to an average at harvest of 38.8% (range 32.9% to 43.2%) for low seed and of 29.3% (range 19.2% to 39.2%) for high seed. For CD8^+^ T-cells, this increase was from 1.5% (range 1.2% to 2.0%) to 42.9% (range 30.5% to 51.4%) for the low seed and of 25.5% (range 20.0% to 33.3%) for high seed products. Co-expression of these markers was very low, generally < 1% of CD4^+^ or CD8^+^ T-cells, as shown in Table 2. The highest levels were seen for PD-1 and Tim-3 co-expression on CD4^+^ T-cells from both low and high seed products, with mean expression levels of 7.7% (range 1.7% to 15.3%) and 6.8% (range 1.4% to 10.0%) respectively.

**Table 2:** Co-expression of exhaustion markers

T-cell source	Percentage of T-cells co-expressing LAG-3, PD-1 and Tim-3							
	CD4^+^ T-cells				CD8^+^ T-cells			
	LAG-3/PD-1/Tim-3	LAG-3/PD-1	LAG3/Tim-3	PD-1/Tim-3	LAG-3/PD-1/Tim-3	LAG-3/PD-1	LAG-3/Tim-3	PD-1/Tim-3
Pre	0	0	0.1 (0.07–0.23)	0	0.1 (0.03–0.21)	0	0.1 (0.05–0.29)	0.1 (0.03–0.21)
LS	0.1 (0.07–0.20)	0	0	7.7 (1.66–15.30)	0.2 (0.01–0.61)	0	0.2 (0.12–0.43)	2.0 (1.34–3.44)
HS	0.2 (0.09–0.31)	0	0.1 (0.10–0.21)	6.8 (1.42–10.00)	0.3 (0.14–0.40)	0	0.4 (0.22–0.64)	1.3 (0.90–1.89)

Mean percent and ranges (parentheses) for T-cells from 5 donors are shown. Values less than 0.1% are reported as 0

*PRE* pre-expansion PBMCs, *LS* low seed, *HS* high seed

Expression levels measured by mean fluorescence intensity (MFI) of these 3 exhaustion markers were also assessed (Fig. 4f). For all harvested cell products, LAG-3 had very low expression that did not appreciably increase following cell expansion. PD-1 expression on CD8^+^ T-cells also did not increase following expansion, whereas on CD4^+^ T-cells, the marker increased slightly. For Tim-3, all harvested products had increased expression levels in both CD4^+^ and CD8^+^ T-cell populations.

### Functionality of T-cells expanded in the Quantum system

**Fig. 5:**
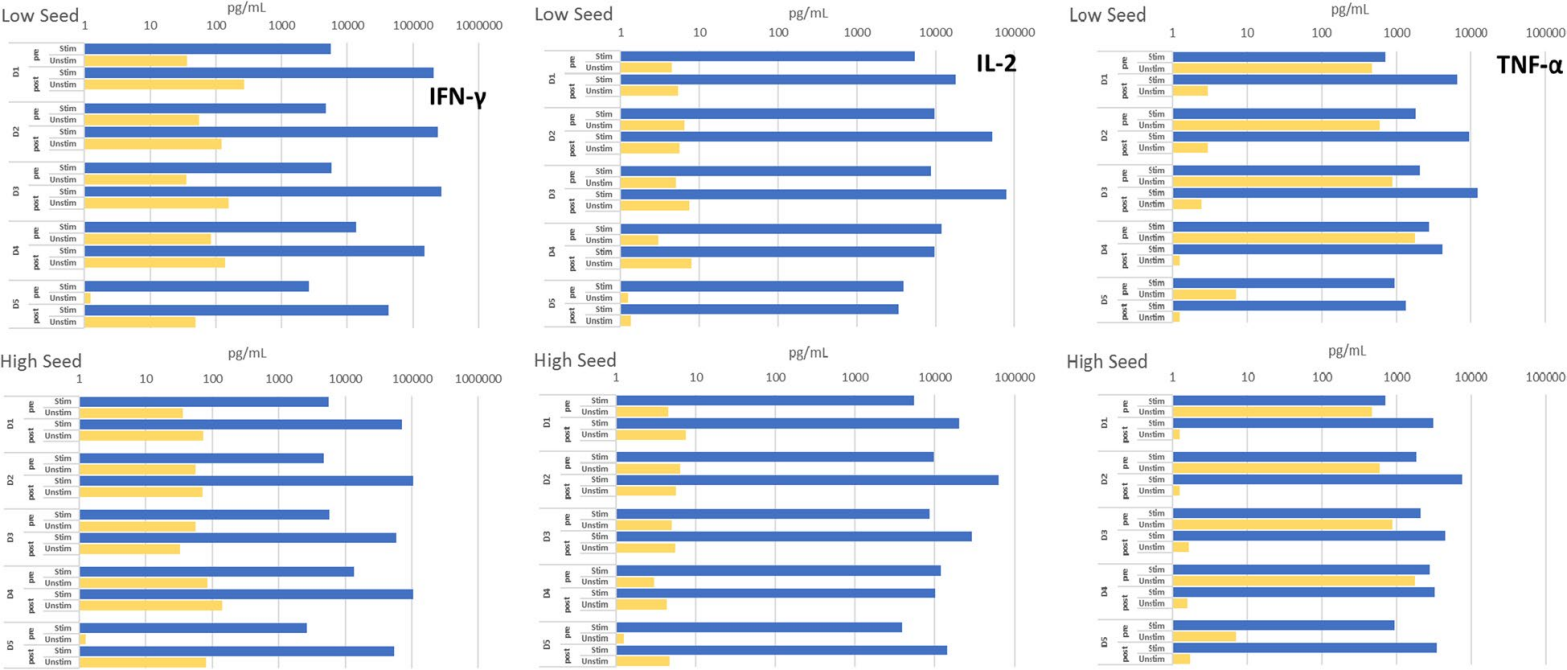
Cytokine secretion profiles for T-cells expanded in the Quantum system. Secretion of IFN-γ, IL-2 and TNF-α from cell populations pre-seeding (pre) and after harvest following restimulation (post) for T-cells expanded from PBMCs of 5 healthy donors. Cells were either not stimulated or were restimulated overnight with anti-CD3/CD28 Dynabeads and cytokine concentrations in the supernatants were assayed by MesoScale multiplex cytokine assay

Harvested T-cells from all donors secreted high amounts of the pro-inflammatory cytokines, IFN-γ, IL-2 and TNF-α, following polyclonal restimulation (Fig. 5), with similar trends for low and high seed cultures. There was a high background of TNF-α produced by pre-expansion PBMCS in the absence of stimulation, but harvested T-cell products had greatly reduced background levels and very high ratios for stimulated versus non-stimulated conditions. Samples of all harvested T-cells were debeaded by passage over magnetic columns to remove residual Dynabeads prior to restimulation, but the cytokine secretion profiles of these samples were not different from those of the non-debeaded samples (data not shown).

### Usage of medium and cytokines in T-cell expansion in the Quantum system

**Fig. 6:**
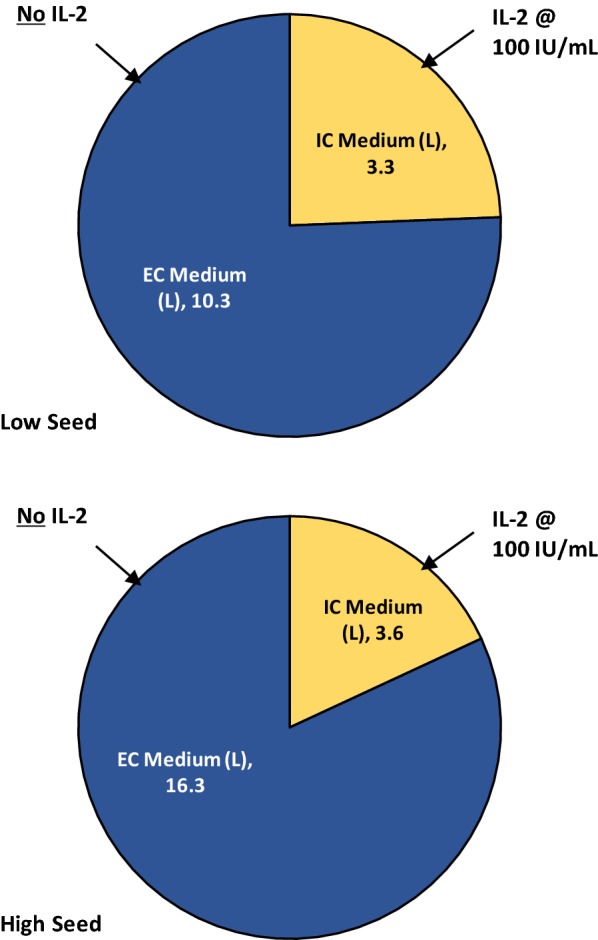
Culture medium and cytokine usage in Quantum system T-cell expansions

The total usage of medium for T-cell expansion from high and low seed cultures is illustrated in Fig. 6. For high seed expansions, a total of 19.9 L was used although only 3.6 L of medium was used on the IC side of the bioreactor and IL-2 was only added to this compartment and not to the EC side of the bioreactor. The total consumption of IL-2 per expansion was 0.36 × 10^6^ IU for production of an average of 23.5 × 10^9^ T-cells, whereas 2 × 10^6^ IU would have been required if all 19.9 L had included IL-2 at 100 IU/mL. Similarly, low seed expansions, producing an average of 14.2 × 10^9^ T-cells, used 13.6 L of medium but only 0.33 × 10^6^ IU IL-2 as only 3.3 L of medium on the IC side of the bioreactor contained the cytokine.

## Discussion

The tremendous potential and promise of cellular therapies for treatment of cancer are tempered by the challenges associated with production of cell products in therapeutic doses under GMP requirements for consistency, sterility and quality. In this report, we show that the Quantum system is a promising expansion platform for deployment in the cell therapy space, where it could be utilized in manufacturing processes in which end users would validate their entire process for their intended use. Cell culture expansion was started directly in the Quantum system with no requirement for a flask culture intermediary, thus reducing the risk of contamination. The risk was further reduced by the use of functionally-closed tubing systems in which cell inlet bags and media bags were sterile-welded directly on to the Quantum system disposable sets.

The Quantum system has previously been described as a robust platform for expanding adherent cells such as adult stem cells [4–8] and, more recently, preliminary results for expansion of non-gene-modified T-cells were reported [9]. We now show that the Quantum system supports consistent expansion of T-cells from apheresis collections from multiple healthy donors, yielding numbers of CD3^+^ T-cells in clinical dose ranges (approximately  13 × 10^9^ to 25 × 10^9^ T-cells per expansion run depending on numbers of cells seeded) in a short time frame of 8 to 9 days and with consistent viability, fold expansion and doubling times. The specific protocols and instrument settings employed to achieve these high cell numbers have been previously described in detail and include accelerated feeding for improved nutrient supply, increased oxygen supply and increased lactate removal [9]. Previously reported T-cell expansion in the Quantum system was 500-fold; the present study demonstrates 760- and 1300- mean fold expansions depending on the starting cell numbers. The reason for the difference in expansion efficiency may be the use of different medium formulations: PRIME-XV [FUJIFILM Irvine Scientific] was used in the current report whereas TexMACS™ [Miltenyi Biotec] was used in the earlier report. Despite rapid and high expansion, all harvested products retained functionality upon restimulation as measured by IFN-γ, IL-2 and TNF-α secretion, although there were some differences among the donors in the T-cell phenotypes generated as discussed further below. There were also some differences between T-cell products from low and high PBMC seeding numbers. These different starting cell numbers were used to investigate the versatility of the Quantum system, and overall products of low and high seedings were functional and had similar frequencies of T-cell memory subsets. Expansion occurred from input cell numbers as low as 7 × 10^6^ CD3^+^ T-cells in a total of 30 × 10^6^ PBMCs (see Table 1, donor 5). This is an important consideration for expansion of T-cells from patients, particularly pediatric patients, who may have low numbers of circulating CD3^+^ T-cells and for whom apheresis blood collection may be restricted or challenging. We are continuing to investigate the lower limits of cell numbers that can be used to initiate T-cell expansion in the Quantum system. For high seed cultures, nutrients may have been limiting toward the end of the T-cell expansions, as suggested by the more rapid decline of lactate generation rates in high seed cultures at later timepoints. Increasing the feed rates in the Quantum system beyond those applied here could have the potential to result in greater T-cell harvest yields, a subject which we are currently investigating.

Current autologous CAR T-cell dose ranges are lower than the T-cell yields reported here for the Quantum system (maximum adult doses for axi-cel and tisagenlecleucel are 2.0 × 10^8^ and 6.0 × 10^8^ cells respectively) [YESCARTA™/axi-cel and KYMRIAH™/tisagenlecleucel prescribing information]. This suggests that the Quantum system may have the potential to reduce the culture time required for preparation of autologous CAR T-cell doses because of the rapid cell expansion that the system can support (see Fig. 2). This may be crucial for both the stage of T-cell differentiation required for efficacy and for expedient treatment of patients with rapidly-advancing disease [10]. Since cell numbers can be monitored during expansion via sterile sampling from the IC loop, cultures can potentially be harvested upon attaining target cell numbers with the desired phenotype, similarly to methodology that has been described for axi-cel [3].

The CD4/CD8 ratios of harvested T-cells differed somewhat among individual donors and differed between cell expansion products from the same donor initiated with either low or high seeding numbers, although total numbers of doublings were very similar. In general, CD8^+^ T cells expanded preferentially in low seed cultures and CD4^+^ T cells expanded more in high seed cultures. This may suggest that IL-2 was limiting for the high seed cultures. Lower exogenous IL-2 availability could bias towards CD4^+^ T-cells, which generally secrete higher levels of IL-2 than CD8^+^ T-cells. Although there is currently no definitive evidence that there is a preferred ratio of CD4^+^ to CD8^+^ T-cells for optimal therapeutic efficacy and safety of CAR T-cell products [11–14], defined ratios have been shown to elicit superior efficacy in preclinical models [15]. However, in the ZUMA-1 clinical trial of axi-cel, averages of 57% CD8^+^ and 43% CD4^+^ T-cells comprised the product [14], and for tisagenlecleucel, similar ratios of CD4^+^/CD8^+^ T-cells were present in the products for both responders and non-responders, with expansion of CAR T-cells in vivo occurring independently of this ratio [13]. These clinical data suggest that the CD4/CD8 ratios in T-cell products produced here in the Quantum system would be applicable for immunotherapy.

CD8^+^ T-cells harvested from the Quantum system displayed less-differentiated phenotypes of TN and TCM and CD4^+^ T-cells were predominantly TCM. These phenotypes are currently believed to be the  most potent subsets for adoptive immunotherapy in hematologic cancers, with superior durability in vivo and enhanced proliferative capacity [15–17] although unfractionated CAR T-cells are being perfused in most clinical trials [18], and in patients treated with tisagenlecleucel [13]. In addition, less-differentiated T-cell memory phenotypes had strong correlations with responses to therapy in chronic lymphocytic leukemia (CLL) patients, and late memory and TE phenotypes were correlated with poor outcomes in the same patient group [12].

All T-cell products derived from the Quantum system had low frequencies of cells bearing the exhaustion markers LAG-3 and PD-1 and these markers did not increase during expansion. In contrast, Tim-3 expression frequency increased on all products following expansion. Although Tim-3 has been shown to have a role in exhaustion of T-cells, it may also have costimulatory capacity and its exact mechanistic functions are likely not yet fully elucidated [19–21]. Moreover, co-expression of Tim-3 with other exhaustion markers on T-cell products was low and, since T-cell dysfunction is generally associated with co-expression of inhibitory markers and receptors [22–24], it is unlikely that T-cells harvested from the Quantum systems would be dysfunctional. In addition, following restimulation in vitro, all harvested T-cell products responded potently with Th1-type cytokine secretion, indicating that these populations were functional and not exhausted. The phenotypes and differentiation pathways of T-cells and gene-modified T-cells expanded in vitro are influenced by many factors including the cytokines used for expansion [25], disease status of the patient [10, 25], duration time of cells in culture [10] and composition of the culture medium used [26]. These factors are currently being evaluated in the Quantum system to better understand the system’s versatility and advantages, particularly with regard to the growth of CAR T-cells.

Other semi- and fully-automated expansion technologies are available for growth of T-cells for immunotherapy, e.g. the Xuri™ W25 and WAVE Cell Expansion systems (GE Healthcare Life Sciences) [27, 28], Gas Permeable Rapid Expansion (G-Rex^®^) devices (Wilson Wolf Corporation, St. Paul, MN) [29, 30] and the CliniMACS Prodigy^®^ (Miltenyi Biotec) [31–34]. With all these systems, as well as with the Quantum system, the goals are to provide functionally-closed systems for T-cell growth and to obtain high cell yields while minimizing risk of contamination. While these other systems have distinct attributes applicable for different cell expansion settings, published cell yields encompass those presented here. Reported yields from the Prodigy are lower than the Quantum system [31–34], with ranges up to 6 × 10 ^9^ CAR T-cells when healthy donor cells were used [34]. G-Rex reported yields are similar to Quantum system yields, with up to 20 × 10^9^ T-cell receptor (TCR)-engineered T-cells obtained with products derived from cancer patients as well as healthy donors [30]. Yields for CAR T-cells from the WAVE have been reported to be similar to the Quantum system, with ranges up to 24 × 10^9^ for cells derived from CLL patients [28], although with higher yields of 44 × 10^9^ cells reported for tumor infiltrating lymphocytes (TILs) [27].

A unique feature of the Quantum system is the dual loop system that allows for partitioning of different formulations and volumes of medium to the IC and EC fluid loops, as illustrated herein. In addition, the dual loop design has the potential to reduce use of cytokines and growth factors such as IL-2, since these can be preferentially added to the relatively small volume of IC medium only, with nutrient and gas supply provided from the EC side. For the T-cell expansions in the Quantum system described here, although a total of 20 L medium was consumed in the high seed feeding protocol, which generated an average of 23.5 × 10^9^ T-cells, only 3.6 L of medium on the IC side of the bioreactor contained IL-2 (see Fig. 6), reducing the amount of IL-2 required by 82%. Considering that in expansion of TILs or TCR-engineered cells for autologous immunotherapeutics, use of 3000 U/mL IL-2 has typically been reported with other bioreactor systems [27, 30, 35], it is clear that the use of the Quantum system could result in substantial savings on the costs of this cytokine.

TILs are currently being tested for treatment of solid tumors and have been infused at extremely high doses to date. For example, Tran et al. infused a dose of 1.48 × 10^11^ TILs to a patient with metastatic colorectal cancer [35] and Zacharakis et al. described infusion of 8.2 × 10^10^ TILs to a patient with chemorefractory hormone receptor-positive metastatic breast cancer [36]. In both cases, the authors reported durable regression of metastatic disease. With the current configuration, it would require several Quantum system expansions to generate T-cell harvest numbers in dose ranges compatible with requirements for TILs. However, substantially higher T-cell numbers have been harvested using medium supplemented with additional ingredients such as albumin and human serum, indicating the potential for the Quantum system to support cell yields as high as approximately 40 × 10^9^ T-cells (see Additional file 2). Moreover, as discussed above, increasing feed rates to the bioreactor may also result in higher T-cell harvest yields.

Allogeneic universal CAR T-cell products, derived from healthy donors, are also being developed as potential future cancer immunotherapies. This approach has advantages over autologous therapies in terms of standardization in manufacturing processes, removal of inter-patient variability and removal of the impact of patient health on the final product [37, 38]. Although allogeneic cell therapeutic doses are likely to be in similar ranges to autologous products, the ability to expand donated products to very high numbers would be necessary to create master cell banks and working cell banks for off-the-shelf use. The Quantum system has the capacity to support allogeneic cell therapy manufacturing by generation of T-cell products in the range of 23 × 10^9^ T-cells from a single expansion using defined, serum-free medium as reported here. Based on these yields of T-cells, a single apheresis mononuclear cell collection from a healthy donor yielding 4.3 × 10^9^  mononuclear cells (Terumo BCT internal data for the Spectra Optia^®^ apheresis system) could be expanded via scale-out in Quantum systems to allow production of almost 4000 doses of allogeneic T-cells, based on the current maximum dosing for tisagenlecleucel in B-ALL [13]. If dosing were in range for the maximum doses of tisagenlecleucel recently prescribed for DLBCL (6 × 10^8^ CAR^+^ T-cells; KYMRIAH/tisagenlecleucel prescribing information), approximately 1500 doses could potentially be manufactured from a single donor. In addition, alternative allogeneic effector cells for use as universal cell therapies such as NK cells, NKT cells and γδ T-cells, which do not cause graft-versus-host-disease [39], are likely to be amenable to expansion in the Quantum system following similar protocols to those described here.

## Conclusion

This report highlights use of the Quantum system as a promising platform for growth and expansion of T-cells as immunotherapeutics to target cancer and could have wide applicability for both autologous and allogeneic cellular therapies. Large quantities of functional T-cells at clinical dosing scale were consistently produced within a short timeframe. Future directions for this platform include evaluation for expansion of other types of suspension cells and for gene-modified T-cells in particular.

## Additional files

**Additional file 1.** Quantum system custom task default settings. Tables show the custom tasks, settings and feed schedules that were used for T-cell expansions in the Quantum system.

**Additional file 2.** T-cell expansion in the Quantum system using alternative types of medium. Graphs show T-cell expansions in the Quantum system using alternative media formulations from the one described in the main manuscript and illustrate that the Quantum system can support cell yields as high as 40× 10^9^ T-cells.

## Data Availability

The data that support the findings of this study are available from the corresponding author upon reasonable request.

## Abbreviations

B-ALL: B-cell acute lymphoblastic leukemia
CAR: chimeric antigen receptor
CES: Cell Expansion System
CLL: chronic lymphocytic leukemia
CV: coefficient of variation
DLBCL: diffuse large B-cell lymphoma
DS: cell doublings
EC: extracapillary
FDA: US Food and Drug Administration
h: hours
HF: hollow fiber
IL-2: interleukin 2
IC: intracapillary
MFI: median fluorescence intensity
min: minutes
PBMCs: peripheral blood mononuclear cells
PBS: phosphate buffered saline
Quantum system: Quantum Cell Expansion System
r/r DLBCL: relapsed or refractory diffuse large B-cell lymphoma
TCPS: tissue culture polystyrene substrates
TCM: central memory T-cells
TE: effector T-cells
TEM: effector memory T-cells
TILs: tumor infiltrating lymphocytes
TN: naïve T-cells
