# Supplementary material for: Large-scale expansion and characterization of CD3+ T-cells in the Quantum® Cell Expansion System
Source: J Transl Med. 2019 Aug 7;17:258. doi: 10.1186/s12967-019-2001-5 (PMC6686483; doi:10.1186/s12967-019-2001-5)
Supplement: Supplementary file 1 — Additional file 1. Quantum system custom task default settings. Tables show the custom tasks, settings and feed schedules that were used for T-cell expansions in the Quantum system. [file 12967_2019_2001_MOESM1_ESM.docx]

**Additional file 1**

**Table 1 A and B. Quantum System Custom Task Default Settings**

Tables 1 and 2 show the custom tasks and settings that were used for T-cell expansions in the Quantum system.

Complete: medium containing IL-2

Base: medium without IL-2

**Table 1A. Custom 1 and Custom 2**

**Table 1B. Custom 3 and Custom 4**

**Table 2 A and B: Quantum System Expansion Schedule**

Tables 2 A and B show the feed schedules that were used for T-cell expansions in the Quantum system.

**Table 2A. Low Seed**

**Table 2B. High Seed**
