# Supplementary material for: Large-scale expansion and characterization of CD3+ T-cells in the Quantum® Cell Expansion System
Source: J Transl Med. 2019 Aug 7;17:258. doi: 10.1186/s12967-019-2001-5 (PMC6686483; doi:10.1186/s12967-019-2001-5)
Supplement: Supplementary file 2 — Additional file 2. T-cell expansion in the Quantum system using alternative types of medium. Graphs show T-cell expansions in the Quantum system using alternative media formulations from the one described in the main manuscript and illustrate that the Quantum system can support cell yields as high as 40× 109 T-cells. [file 12967_2019_2001_MOESM2_ESM.docx]

**Additional file 2**

**T-cell expansion in the Quantum System using alternative types of medium**

PBMCs were incubated with anti-CD3/CD28 DynaBeads, then seeded in the Quantum system bioreactor at either 30 × 10^6^ (A; low seed) or 80 × 10^6^ to 120 ×10^6^ (B; high seed) PBMCs. Five healthy donors were used for low seed expansions and three healthy donors were used for high seed expansions. Numbers of cells indicate total mononuclear cells at various timepoints enumerated from samples taken from the IC loop following mixing of the entire IC compartment. Final cell numbers are for products enumerated following automated harvest from the Quantum system.

Types of medium used were: RPMI 1640 basal medium (Corning catalog number 15040-CM) supplemented with 5% fetal bovine serum (FBS; VWR/Seradigm catalog number 97068-085 or Hyclone catalog number SH30070.03); TexMACS - animal component-free medium (TexMACS^TM^ GMP medium, Miltenyi Biotec catalog number 170-076-306); RPMI Advanced - RPMI 1640 basal medium containing proteins AlbuMAX^®^II (lipid-rich bovine serum albumin), human transferrin and recombinant insulin full chain (Gibco^®^ catalog number 12633012), and supplemented with 2% human AB serum (HS; Corning/VWR catalog number 35-060-CI); X-VIVO^TM^ 15 – serum-free medium (Lonza); X-VIVO-15 supplemented with 2% HS; Iscove’s modified Dulbecco’s medium (IMDM; Lonza^TM^ BioWhittaker^TM^ catalog number 12726Q) supplemented with 5% FBS; PRIME-XV research grade medium (FUJIFILM Irvine Scientific) supplemented with 2 % HS. All types of medium were additionally supplemented with recombinant human IL-2 improved sequence (Miltenyi Biotec) at a final concentration of 100 IU/mL.

Other methods and reagents are as described in the Materials and Methods section of the manuscript. Error bars represent one standard deviation from the mean.

**Additional Results**

**Figure S1 A and B. T-Cell expansion in the Quantum system using different types of medium**

Growth curves for T-cells expanded in the Quantum system using different types of cell culture medium.

A) Low seed: RPMI 1640 + 5% FBS– graph shows the average of 5 expansions with 2 different donors; TexMACs – one expansion with a single donor; RPMI Advanced + 2% HS – one expansion with a single donor; X-VIVO 15 – one expansion with a single donor; X-VIVO 15 + 2% HS – one expansion with a single donor (the same donor used with X-VIVO 15); IMDM + 5% FBS –average of 2 expansions with 2 different donors; PRIME-XV + 2% HS – average of 2 expansions with 2 different donors.

B) High seed: TexMACS – graph shows the average of 3 expansions with 2 different donors (one donor was repeated); RPMI Advanced + 2% HS – average of 4 expansions with 3 different donors (one donor was repeated).

Various types of medium and supplements were evaluated for their capacity to support T-cell expansion in the Quantum system. In particular, the maximum cell capacity of the bioreactor was assessed by addition of supplements such as FBS and HS.

Figure A indicates that different types of medium and supplements influence cell yield and growth kinetics in the Quantum system when starting from a low seeding density. In general, the addition of FBS or HS increased the cell yield substantially, e.g. addition of 2% HS to X-VIVO 15 medium more than doubled the cell yield from 11.1 × 10^9^ to 27.4 × 10^9^ total cells.

At a high seeding density (Figure B), using RPMI advanced medium additionally supplemented with 2% HS, an average yield of 39.8 × 10^9^ cells (range 31.7 to 43.6 × 10^9^ cells) with 99 % CD3^+^ T-cells (range 96.6 % to 100.0 %) was obtained from the Quantum system in 8 days. This suggests that with minimal scale-out of the Quantum systems, concentrations and yields of T-cells compatible with current requirements for TILs could be achieved.

**A. Low Seed**

**B. High Seed**
